# Reputation-surveillance model of mate guarding: community size and religious veiling

**DOI:** 10.1017/ehs.2024.40

**Published:** 2024-10-30

**Authors:** Farid Pazhoohi

**Affiliations:** School of Psychology, University of Plymouth, Plymouth PL4 8AA, UK

**Keywords:** Community size, veiling, mate guarding, cultural evolution

## Abstract

The mate guarding theory of conservative clothing posits that veiling reduces women's physical allure and sexual attractiveness, thereby diminishing men's attraction towards them and deterring potential rivals for a woman's partner. This theory argues that the importance of veiling is influenced by ecological factors in a way that it is of higher importance to control women's sexuality in harsher environments to secure paternal investment. A prediction of this theory is that the importance of veiling should be influenced by community size, where individuals’ reputations, specifically men's, might have different weightings, and their perceived sense of controlling a partner's activity may differ. Using pre-existing data from seven countries encompassing over 9000 individuals, the current study explored the association of town size and importance of veiling for women. Results showed a U-shaped relationship where in small towns and large cities, individuals, specifically men, give more importance to the veiling of women. This finding not only has multiple implications in terms of the effect of community size on male policing behaviours of women and sexual restrictions, but it also might point to a wider relationship regarding the association of community size and moral values.

## Graphical abstract

**Figure:**
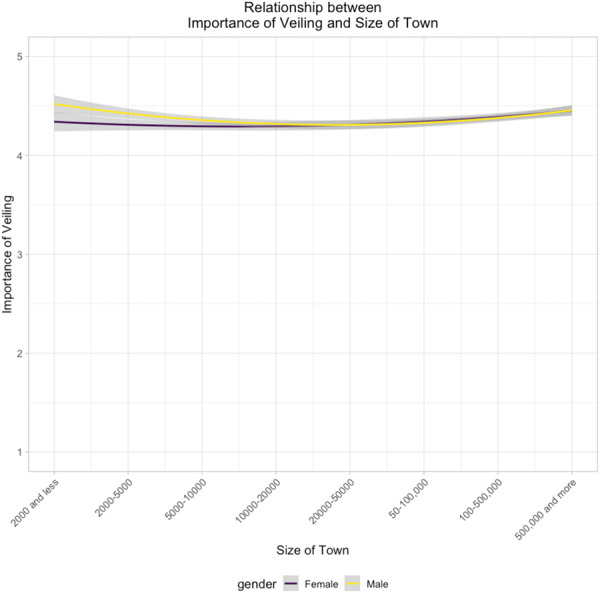

**Social media summary:** The study shows a U-shaped link between town size and veiling, highlighting how community size impacts sexual policing.

## 1. Introduction

Those in towns, are always wrapped up in a large white sheet, which covers them to their feet, and completely hides their figure. They are enabled to see by means of a net-work in the white hood, which covers their head … In the country they go unveiled, and are under no other restraint among people of their own camp or village, than what is imposed by the general opinion, that it is indecent to associate with the men … the chastity of the country women, and particularly of those of the shepherds, is a theme of praise to all people acquainted with their manners. (Elphinstone, 2011)

Veiling, is an ancient practice upheld by religious beliefs, social norms and legal frameworks, involves women covering parts of their bodies, such as the head, face or entire body, with specific clothing or fabric and has taken various forms throughout history and across different cultures. It has been practised by ancient civilisations such as the Assyrians, Greeks and Persians (Cairns, 2002; Jastrow, 1921; Scarce, 1975) and has been adopted by Middle Eastern-rooted religions, including Judaism, Christianity and Islam (Bronner, 1993; Chico, 2000; Tariq, 2013). Historically, veiling was a symbol of piety, purity and class, and it was forbidden for prostitutes and slaves (Graeber, 2012; Jastrow, 1921; Lerner, 1986). In Judaism, head coverings were traditionally worn by married women as a symbol of modesty and marital status. In Christianity, especially within certain sects like Catholicism and Eastern Orthodoxy, veils were used during religious services to signify piety and humility. The spread of Islam in the seventh century CE facilitated the adoption of veiling across the Middle East, North Africa and parts of Asia. While the practice significantly diminished in Europe from medieval times (Burghartz, 2015; Koslin, 2008), it continues to be frequently observed in the Islamic world today. Over the last century, veiling has become primarily associated with Islamic faith and identity (Ruby, 2006).

### 1.1. The mate guarding theory of veiling

In a series of papers, Pazhoohi and colleagues, adopting an evolutionary adaptive perspective, introduced the mate guarding theory of conservative clothing (Pazhoohi, 2016, 2023; Pazhoohi & Hosseinchari, 2014; Pazhoohi et al., 2017a). This approach extends beyond the social and personal aspects of veiling and hijab to explore the adaptive reasons behind this practice. The theory proposes that veiling decreases women's attractiveness, serving to prevent mate poaching and allow the restriction of women's sexuality by male relatives. In addition to studies demonstrating the effect of veiling on decreasing the attractiveness ratings of women (Jordan et al., 2020; Mahmud & Swami, 2010; Pazhoohi & Hosseinchari, 2014; Sheen et al., 2018), eye-tracking research has shown that there is less attention focused on the body curves of women wearing conservative vs. liberal clothing, thereby lowering attractiveness ratings (Pazhoohi et al., 2017b). Conservative religious clothing also reduced male encounters in real-world settings; motorists were more inclined to offer rides to women dressed in liberal attire compared with those in conservative clothing (Pazhoohi & Burriss, 2016).

The mate guarding theory of veiling also suggests that religious conservative clothing, far from being merely a traditional or aesthetic choice, is a complex adaptive behaviour that responds to specific ecological pressures (Pazhoohi et al., 2017a). This adaptation can be understood within the framework of socio-biological theories, where cultural practices evolve to mitigate reproductive and survival challenges faced by communities (Fitouchi et al., 2023; Luberti et al., 2023; Moon, 2021; Van Slyke & Szocik, 2020). For example, Pazhoohi and colleagues (Pazhoohi et al., 2017a; Pazhoohi & Kingstone, 2020) have shown that environments characterised by high mortality risks and uncertain child survival outcomes significantly amplify the cultural endorsement of veiling. In such environments, where significant paternal investment is necessary owing to scarcity of resources, greater effort is directed towards controlling and guarding mates against infidelity. In addition to the increased importance of bi-parental care in such environments, the frequent and often extended absence of men from the settlement in historically pastoral societies intensifies the importance of restrictions on women's mobility and sexual behaviours (Becker, 2024). Additionally, research indicates sex differences in veiling bias, with men and mothers of sons showing more support (Blake et al., 2018, 2021; Pazhoohi & Kingstone, 2020). Research not only indicates that men are more supportive of veiling than women (Blake et al., 2018, 2021; Pazhoohi & Kingstone, 2020), but also that this support intensifies in environments where child survival is at greater risk. Importantly, the results showed that across 25 Muslim countries and various geographical regions, Muslim men displayed a higher level of support for veiling compared with Muslim women (Pazhoohi & Kingstone, 2020).

### 1.2. Community size and veiling: reputation and surveillance

Overall, the mate guarding theory of veiling suggests that veiling serves as a strategy for men to maintain their partners and deter mate-poaching by making it difficult for potential rivals to assess the attractiveness of their partners, crucially in environments where paternal certainty impacts child survival. Considering the adaptability of cultural practice of veiling as a function of ecological–biological factors (Pazhoohi et al., 2017a; Pazhoohi & Kingstone, 2020), the practice of veiling should be contextualised within the size of the community, as this can affect how much weight is placed on individuals’ reputations, particularly those of men. Cross-cultural research indicates that the perception of reputation vulnerability and ethical behaviour is significantly influenced by community size, as members of small rural communities perceived their reputation as more vulnerable compared with those in large cities and exhibited a reduced willingness to engage in unethical actions (Danielson et al., 2023). Regardless of religiosity and fear of divine punishment, people in small communities, where reputational concerns are more prominent, donated more and were more cooperative than those in larger communities (Ge et al., 2019). Cuckoldry not only poses the evolutionary risk of a man failing to pass his genes to the next generation and inadvertently investing resources in another man's offspring, but indications of a partner's infidelity can also severely impact his future mating opportunities, as well as diminish his social standing and reputation (Buss, 2002; Wilson & Daly, 1992). Therefore, one can argue that in small communities, veiling is of high importance, as it not only signals a woman's modesty and fidelity but also a man's vigilance and control over his family's reputation, indirectly safeguarding his genetic legacy and social standing.

Additionally, the extent to which men feel they can control their partner's activities varies with community size, largely owing to differences in anonymity and social monitoring. In smaller communities, close-knit relationships and high social cohesion mean that individuals’ actions are more visible and subject to communal scrutiny, reinforcing social norms and making control easier. Conversely, in larger communities, increased anonymity and a greater number of social interactions reduce the effectiveness of informal social controls, allowing more freedom from community oversight. This anonymity diminishes social pressures, making it more challenging for men to monitor and control their partners’ activities. Accordingly, while reputation is of lower concern in larger communities owing to the anonymity afforded by larger populations (Danielson et al., 2023; Suzuki & Akiyama, 2005), monitoring a partner's behaviour might be harder, leading to alternative strategies for ensuring fidelity and mate guarding. In other words, in large cities, the difficulty of surveillance leads to a resurgence in the emphasis on veiling. This resurgence stems from men's decreased ability to monitor their partners’ activities, thus resorting to veiling as a preventive measure against infidelity and to signal their concern for maintaining control over their relationships. Indeed, ecological research has shown that conservative veiling decreases approaches by male motorists (Pazhoohi & Burriss, 2016).

The current study explores the potential relationship between community size and the importance of veiling. Specifically, using data from more than 9000 individuals across seven Muslim countries from the World Value Survey dataset, the importance of veiling is investigated as a function of town size, while controlling for the sex of participants, the importance of religion and income level.

## 2. Methods

Data were acquired from the World Value Survey's Longitudinal Multiple-Wave dataset (Inglehart et al., 2022). Specifically, Wave 4 (1999–2004) was utilised, as it has been the only wave to include a variable for the importance of veiling. All participants were over 18 years of age at the time of participation and identified Islam as their faith. Only those participants for whom data on the importance of veiling or the size of the town were available were included in the analysis (*N* = 10,016). After excluding individuals without information on income level and the importance of religion, the final sample comprised 9069 participants from seven countries: Algeria, Bangladesh, Egypt, Indonesia, Jordan, Nigeria and Saudi Arabia (see Table 1).

**Table 1.** Sample size, age mean, and standard deviation by respondent sex and country

	Male			Female	
Country	*N*	Age, mean (SD)		*N*	Age, mean (SD)
Algeria	510	35.9 (14.3)		502	35.0 (12.6)
Bangladesh	809	36.2 (12.0)		652	30.6 (8.3)
Egypt	1363	40.3 (15.5)		1301	36.4 (13.8)
Indonesia	445	44.8 (12.9)		429	44.4 (13.7)
Jordan	560	38.1 (16.7)		565	34.3 (12.3)
Nigeria	314	31.4 (11.4)		271	30.9 (10.9)
Saudi Arabia	671	33.2 (10.4)		677	32.0 (11.0)

### 2.1. Measures

#### 2.1.1. Sex

The sex of the participants was determined based on their responses to question X001 in the survey, which asked about their gender, categorising them as male or female.

#### 2.1.2. Importance of veiling

For the importance of veiling, question D067 (this is IV81 in other codebooks) of the survey was used, which asks about the importance of traits in women (veiling) on a five-point scale from ‘very important’ to ‘not at all important’. These responses were reverse coded.

#### 2.1.3. Income level

For income level measurement, the survey used the income scale (X047), which is a subjective rating of income ranging from 1 (representing the ‘lowest income decile’) to 10 (representing the ‘highest income decile’).

#### 2.1.4. Importance of religion

Variable A006 inquired about the importance of religion in participants’ lives, offering responses ranging from ‘1 – very important’ to ‘4 – not at all important’. These responses were then recoded in reverse, so that a higher number indicated greater importance of religion in daily life.

#### 2.1.5. Size of town

For the size of the town, variable X049 was used, which has values on an eight-point scale and is coded from 1, representing ‘2000 and less’, to 8, representing ‘500,000 and more’.

### 2.2. Data analysis and visualisation

**Figure 1.**
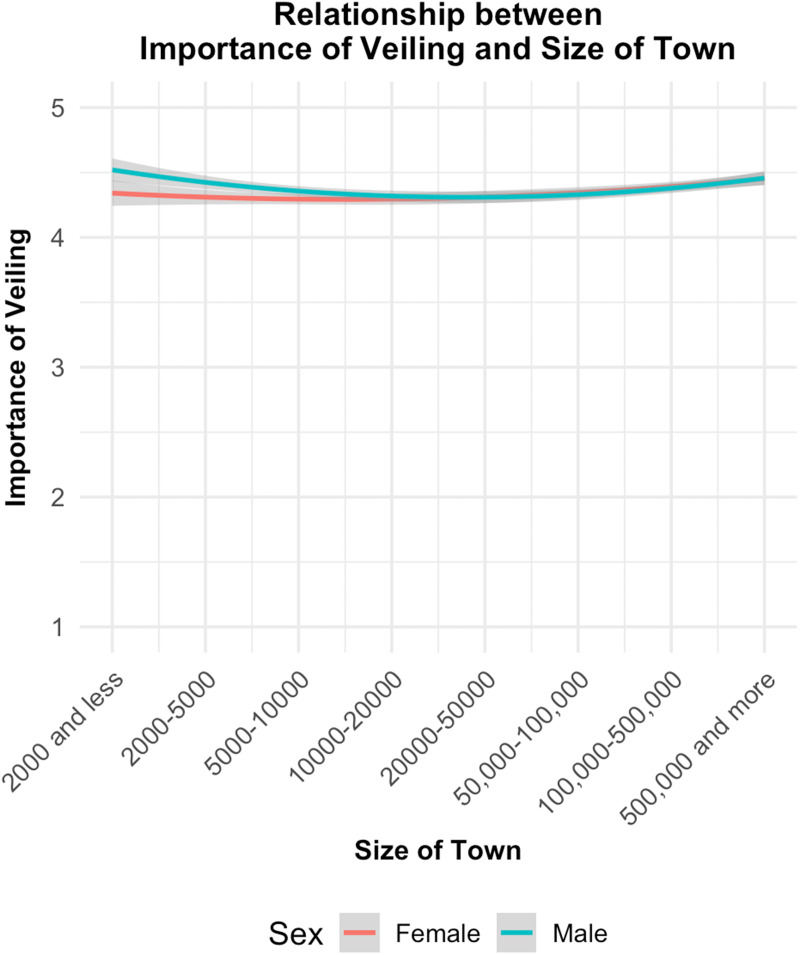
Curve plot for the importance of veiling as a function of size of the town and sex.

The data preprocessing steps involved the exclusion of records with missing values for the variables. All the variables were treated as continuous in the analysis (see Robitzsch, 2020 for a discussion). To visualise the relationship between veiling and the size of the town, a plot was created using ggplot2 in R. Figure 1 illustrates the non-linear relationship between the size of the town and the importance of veiling, with separate lines for male and female respondents. The *x*-axis is labelled with specific population size categories to enhance the clarity and interpretability of the visualisation.

## 3. Results

A linear mixed-effects model was conducted to investigate the effects of sex, the importance of religion, income level, the size of the town and the quadratic term of the size of town on the importance of veiling, with country as a random effect (see Table 2 for the model details; see the supplementary materials for a comparison of the model's fit with a non-quadratic model). The analysis was based on a dataset comprising both men and women (*N* = 9069 observations) across seven countries. The results showed that sex positively predicted the importance of veiling, suggesting that veiling is more important to men than to women. Additionally, there was a positive and significant relationship between the importance of religion and the importance of veiling. However, income level did not significantly predict the importance of veiling. The size of the town showed a significant U-shaped quadratic relationship with the importance of veiling, indicating that the importance of veiling decreases when the town size increases from very small (2000 individuals or fewer) to moderate (20,000–50,000), and then increases with the increase in town size from moderate to large (500,000 and more).

**Table 2.** The fixed and random effects for the model predicting importance of veiling for both men and women (*N* = 9069)

	Estimate	Standard error	*t-*Value	*p-*Value
Intercept	2.70	0.19	14.05	<0.001***
Importance of religion	0.52	0.03	15.99	<0.001***
Sex (male)	0.04	0.02	2.27	0.023*
Income level	0.00	0.00	0.36	0.723
Size of town	−0.18	0.03	−6.40	<0.001***
(Size of town)^2^	0.01	0.01	5.01	<0.001***
Random effects	Variance	Standard deviation		
Intercept (country)	0.10	0.33		
Residual	0.84	0.91		

*Note:* * *p* < 0.05, ** *p* < 0.01, *** *p* < 0.001.

After excluding female participants from the dataset, another model was conducted to investigate the effects of the importance of religion, income level and the size of the town on the importance of veiling for men (*N* = 4672 observations), with country as a random effect (see Table 3 for the model details). The findings of this second model were somewhat similar yet distinct in certain aspects. The importance of religion remained a strong predictor, with a slightly higher coefficient than in Model 1. The size of the town continued to show a significant nonlinear, U-shaped relationship with the importance of veiling, with slightly stronger effects compared with Model 1. However, a similar analysis for female participants (excluding men from the dataset) did not show a curvilinear relationship (see Table 4 and Figure 1).

**Table 3.** The fixed effects for the model predicting importance of veiling only for men (*N* = 4672)

	Estimate	Standard error	*t-*Value	*p-*Value
Intercept	2.74	0.23	11.97	<0.001***
Importance of religion	0.54	0.04	12.91	<0.001***
Income level	0.01	0.01	1.79	0.074
Size of town	−0.24	0.04	−6.37	<0.001***
(Size of town)^2^	0.02	0.01	5.24	<0.001***

*Note:* * *p* < 0.05, ** *p* < 0.01, *** *p* < 0.001.

**Table 4.** The fixed effects for the model predicting importance of veiling only for women (*N* = 4397)

	Estimate	Standard error	*t*-Value	*p-*Value
Intercept	2.73	0.25	10.64	<0.001***
Importance of religion	0.48	0.05	9.48	<0.001***
Income level	−0.01	0.01	−1.51	0.130
Size of town	−0.10	0.03	−2.52	0.011*
Size of town)^2^	0.01	0.01	1.73	0.083
	

*Note:* * *p* < 0.05, ** *p* < 0.01, *** *p* < 0.001.

**Figure 2.**
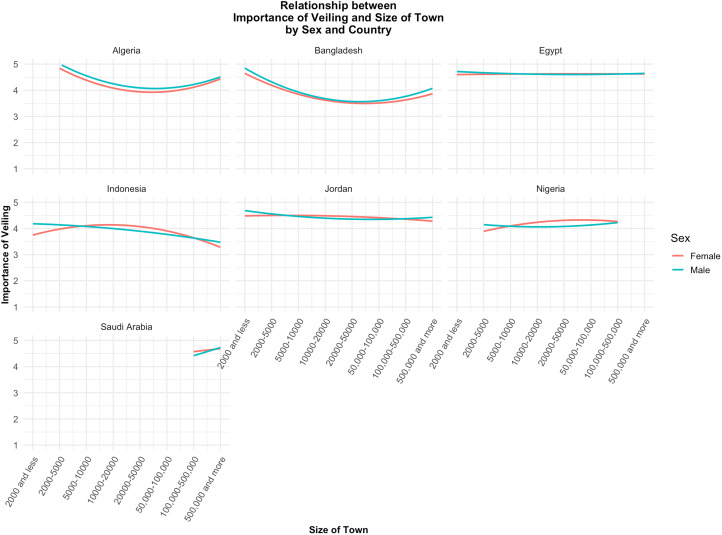
Relationship between importance of veiling and size of town by sex and country.

When conducting separate models predicting the importance of veiling for men in each country, the results revealed distinct curvilinear relationships between the importance of veiling and the size of the town. Specifically, a significant U-shaped relationship was observed for Algeria and Bangladesh, while an inverted U-shaped relationship was identified for Indonesia (see Table S1 and Figure 2).

An additional set of models was conducted that incorporated education as a variable in the analysis. However, the inclusion of education significantly reduced the sample size owing to a substantial number of missing data points, resulting in the exclusion of 1213 individuals from the analysis. The results of these models, including education, are presented in the supplementary material (Tables S3–S5). The results from the model predicting the importance of veiling for both men and women (*N* = 7883) revealed a significant negative relationship between education level and the importance of veiling, indicating that as education increases, the perceived importance of veiling decreases. Income level also had a significant positive effect, suggesting that higher income is associated with a greater emphasis on veiling.

## 4. Discussion

The current study explored how community size influences the perceived importance of veiling across seven Muslim countries, while controlling for the participant's sex, the importance of religion and income. The results showed that the importance of veiling is significantly modulated by sex, with a higher importance placed by men than by women. This is in line with previous research showing that men are more concerned with women's veiling (Blake et al., 2018, 2021; Pazhoohi & Kingstone, 2020), reflecting evolutionary motivations where men's preferences for veiling are tied to concerns over paternity assurance (Pazhoohi et al., 2017a). Moreover, the results showed a positive association between the importance of religion and veiling, while income level did not emerge as a significant predictor of veiling importance. It is noteworthy that in a subsample of participants, where education was included as a variable in the model, both income and education levels emerged as significant predictors of attitudes towards the importance of veiling. Specifically, the analysis revealed that lower levels of education and higher income were associated with greater emphasis on the significance of veiling (Table S3). Further examination indicated that the influence of income was moderated by gender: wealthier men were more likely to consider veiling important, whereas no significant relationship was found between income and the importance of veiling among women (Tables S4 and S5). Conversely, education level was a consistent predictor across genders, with both men and women who had attained higher levels of education placing less importance on veiling.

The results showed a U-shaped relationship between town size and the importance of veiling, highlighting a complex interplay between urbanisation and cultural or religious practices. The findings indicates that in both small towns and large cities, individuals – particularly men – place greater emphasis on the veiling of women. The current paper proposes the Reputation-Surveillance Model of Mate Guarding, positing that the significance of veiling as influenced by community size is determined by two factors: the reputation of a woman's partner and his capacity for surveillance. This proposal needs to be empirically tested, although the archival data used in the current study suggest that mate guarding and policing women's sexuality have a complex relationship with the size of the community. This model suggests that in both small and large communities, policing women's sexuality holds importance, but for distinct reasons in each setting. In small communities, the practice of veiling is emphasised owing to the high value placed on reputation. Since everyone knows each other, a man's ability to monitor his partner is relatively easier, and maintaining a positive reputation becomes crucial. Therefore, veiling is used as a strategy to protect the woman's reputation and, by extension, the man's reputation within the community. This leads to a higher concern for controlling women's appearances as a form of social signalling to deter potential threats to the man's paternity assurance and social standing.

In contrast, the anonymity of large urban centres might make it easier for individuals to engage in behaviours deemed inappropriate without immediate repercussions to their social standing within a smaller community context. This anonymity could raise concerns among men regarding the fidelity of their partners, given the perceived higher chances of encounters that could lead to infidelity. Therefore, in large cities, the emphasis on veiling can also be seen as a mechanism to mitigate these perceived threats, serving as a visible sign of a woman's unavailability and thus a deterrent to potential external romantic interests. This nuanced relationship suggests that the dynamics of veiling are not only a matter of personal or religious choice but are also significantly influenced by the social and cultural environment, especially the urban–rural divide.

Veiling norms affect women differently based on community size. In small communities, women may gain respect and security by adhering to veiling norms, enhancing their social capital and familial honour. However, they face significant social pressure to conform, limiting personal autonomy. In larger communities, where interactions are more diverse and anonymous, women may have more freedom to choose whether to veil. One interesting finding is the divergence in the importance of veiling in small-sized communities between men and women (see Figure 1). While in medium and large-sized communities, such importance aligns for both sexes, men are more concerned with veiling compared with women in small communities (Figure 1). One plausible interpretation might be that women in medium and large-sized communities choose to veil as a form of social identity and as a signal of adherence to norms and conformity. In such communities, where there is a greater diversity of people and potentially more varied social dynamics, veiling can serve as a clear indicator of one's cultural and religious identity. In contrast, in small communities, where individuals are more likely to know each other personally, social cohesion is often maintained through direct, interpersonal relationships rather than through visible symbols of identity. In other words, it might be the case that men and women give similar importance to veiling in medium and large-sized communities, yet for different reasons – men as a means of signalling their partner's unavailability, and women as a sign of social and personal identity and religiosity. In small communities, where veiling might not be as important as a sign of identity for women, men give more importance to such a practice compared with women.

In it noteworthy that in analysing the influence of town size on the perceived importance of veiling among men across different countries, distinct curvilinear patterns emerged. The data revealed a significant U-shaped relationship between town size and the importance of veiling in Algeria and Bangladesh, indicating that veiling is considered more important in both smaller and larger towns, with a decline in medium-sized towns. In contrast, an inverted U-shaped relationship was observed in Indonesia, where the importance of veiling peaked in medium-sized towns and decreased in both smaller and larger towns. Notably, in the remaining countries included in the analysis, no significant association was found between town size and the perceived importance of veiling (see Table S1 and Figure 2). These findings suggest that the socio-cultural significance of veiling is contextually dependent on the specific national settings, highlighting the importance of local demographic and cultural factors in shaping attitudes towards veiling practices. The absence of a similar effect in other countries and its gender-specific nature highlight that these patterns are not universally applicable. Further research should explore whether these effects extend to female respondents and investigate the underlying factors that shape these gendered, context-specific relationships.

A limitation of this study is the lack of direct examination of the relationship between concern for reputation and the importance of veiling, as well as between community size and the difficulty of surveillance. Furthermore, there is no direct evidence to support the idea that the difficulty of surveillance leads to an increased emphasis on veiling. Consequently, future research testing such proposals is warranted. Another limitation of the current study is the limited data points concerning town sizes, which range from very small (2000 individuals or fewer) to very large (500,000 or more). This constraint arises from the reliance on archival data, as is the case in this study.

In summary, the U-shaped relationship observed between community size and the importance of veiling could be attributed to the interplay between reputation management and surveillance capabilities. Results might suggest that in medium-sized communities, where neither factor is as pressing, the practice of veiling diminishes. However, in both extremes of community size, these factors become significant enough to influence the practice, albeit for different reasons. Altogether, the results of this study underscore the complex interplay between community size and cultural practices such as veiling. It suggests that the significance of veiling as a social practice is not merely a reflection of religious or cultural adherence but is also deeply entwined with adaptive psychological mechanisms aimed at mitigating threats to men's social and reproductive interests.

## Supporting information

Pazhoohi supplementary material Pazhoohi supplementary material

## Data Availability

The author will share upon request.

## Supplementary material

To view supplementary material for this article, please visit https://doi.org/10.1017/ehs.2024.40

## Research transparency and reproducibility

The data used in this study are publicly available at https://www.worldvaluessurvey.org/wvs.jsp.
