# Supplementary material for: Reputation-surveillance model of mate guarding: community size and religious veiling
Source: Evol Hum Sci. 2024 Oct 30;6:e37. doi: 10.1017/ehs.2024.40 (PMC11588552; doi:10.1017/ehs.2024.40)
Supplement: Pazhoohi supplementary material [file S2513843X24000409sup001.docx]

Table S1. The Fixed Effects for the Model Predicting Importance of Veiling for Men by Country

| Country | term | estimate | std.error | t value | p value | R_squared | Mean | 2.5 % CI | 97.5 % CI |
| --- | --- | --- | --- | --- | --- | --- | --- | --- | --- |
| Algeria | (Intercept) | 2.16 | 1.03 | 2.10 | 0.04 | 0.10 |  | 0.14 | 4.17 |
|  | Re_importantReligion | 0.97 | 0.15 | 6.38 | 0.00 |  | 3.91 | 0.67 | 1.27 |
|  | incomeLevel | -0.07 | 0.03 | -2.46 | 0.01 |  | 3.08 | -0.12 | -0.01 |
|  | SizeOfTown | -0.67 | 0.29 | -2.34 | 0.02 |  | 5.78 | -1.23 | -0.11 |
|  | SizeOfTown^2 | 0.06 | 0.03 | 2.45 | 0.01 |  |  | 0.01 | 0.11 |
|  |  |  |  |  |  |  |  |  |  |
| Bangladesh | (Intercept) | 2.36 | 0.47 | 5.01 | 0.00 | 0.14 |  | 1.43 | 3.28 |
|  | Re_importantReligion | 0.67 | 0.10 | 6.39 | 0.00 |  | 3.87 | 0.46 | 0.87 |
|  | incomeLevel | 0.02 | 0.02 | 1.09 | 0.28 |  | 4.52 | -0.02 | 0.06 |
|  | SizeOfTown | -0.52 | 0.10 | -5.21 | 0.00 |  | 3.25 | -0.71 | -0.32 |
|  | SizeOfTown^2 | 0.05 | 0.01 | 4.26 | 0.00 |  |  | 0.02 | 0.07 |
|  |  |  |  |  |  |  |  |  |  |
| Egypt | (Intercept) | 4.75 | 0.53 | 9.04 | 0.00 | 0.00 |  | 3.72 | 5.78 |
|  | Re_importantReligion | -0.04 | 0.12 | -0.32 | 0.75 |  | 3.98 | -0.28 | 0.20 |
|  | incomeLevel | 0.00 | 0.01 | -0.04 | 0.97 |  | 5.74 | -0.02 | 0.02 |
|  | SizeOfTown | 0.01 | 0.07 | 0.22 | 0.83 |  | 5.09 | -0.12 | 0.15 |
|  | SizeOfTown^2 | 0.00 | 0.01 | -0.21 | 0.83 |  |  | -0.01 | 0.01 |
|  |  |  |  |  |  |  |  |  |  |
| Indonesia | (Intercept) | 2.86 | 1.52 | 1.87 | 0.06 | 0.09 |  | -0.14 | 5.86 |
|  | Re_importantReligion | 0.20 | 0.38 | 0.51 | 0.61 |  | 3.98 | -0.55 | 0.94 |
|  | incomeLevel | -0.04 | 0.03 | -1.47 | 0.14 |  | 6.18 | -0.09 | 0.01 |
|  | SizeOfTown | 0.39 | 0.14 | 2.89 | 0.00 |  | 3.87 | 0.12 | 0.66 |
|  | SizeOfTown^2 | -0.05 | 0.01 | -3.78 | 0.00 |  |  | -0.08 | -0.02 |
|  |  |  |  |  |  |  |  |  |  |
| Jordan | (Intercept) | 3.19 | 0.74 | 4.30 | 0.00 | 0.01 |  | 1.73 | 4.64 |
|  | Re_importantReligion | 0.32 | 0.18 | 1.79 | 0.07 |  | 3.97 | -0.03 | 0.68 |
|  | incomeLevel | -0.01 | 0.02 | -0.73 | 0.46 |  | 3.47 | -0.05 | 0.02 |
|  | SizeOfTown | 0.05 | 0.09 | 0.60 | 0.55 |  | 4.85 | -0.12 | 0.22 |
|  | SizeOfTown^2 | -0.01 | 0.01 | -0.94 | 0.35 |  |  | -0.03 | 0.01 |
|  |  |  |  |  |  |  |  |  |  |
| Nigeria | (Intercept) | 3.01 | 1.12 | 2.68 | 0.01 | 0.02 |  | 0.80 | 5.21 |
|  | Re_importantReligion | 0.16 | 0.25 | 0.67 | 0.51 |  | 3.93 | -0.32 | 0.65 |
|  | incomeLevel | -0.05 | 0.03 | -1.60 | 0.11 |  | 4.84 | -0.12 | 0.01 |
|  | SizeOfTown | 0.29 | 0.27 | 1.06 | 0.29 |  | 5.22 | -0.25 | 0.82 |
|  | SizeOfTown^2 | -0.02 | 0.03 | -0.77 | 0.44 |  |  | -0.08 | 0.03 |
|  |  |  |  |  |  |  |  |  |  |
| Saudi Arabia | (Intercept) | 3.14 | 0.79 | 3.97 | 0.00 | 0.02 |  | 1.59 | 4.69 |
|  | Re_importantReligion | 0.27 | 0.08 | 3.59 | 0.00 |  | 3.88 | 0.12 | 0.42 |
|  | incomeLevel | 0.00 | 0.01 | -0.35 | 0.73 |  | 5.53 | -0.03 | 0.02 |
|  | SizeOfTown | 0.06 | 0.10 | 0.64 | 0.52 |  | 7.90 | -0.13 | 0.26 |
|  | SizeOfTown^2 |  |  |  |  |  |  |  |  |

**Linear vs Quadratic models**

In comparing the two models predicting the importance of veiling, model 1 and model 2, several key metrics indicate that model 1 is a better fit. Model 1, which includes an additional quadratic term for the size of the town SizeOfTown^2, has 8 parameters compared to the 7 parameters in model 2. The Akaike Information Criterion (AIC) for model 1 is 24201, which is lower than model 2's AIC of 24224, indicating a better fit for model 1. Similarly, the Bayesian Information Criterion (BIC) for model 1 is 24258, compared to 24274 for model 2. The log-likelihood value for model 1 is -12092, higher than the -12105 for model 2, suggesting that model 1 has a higher likelihood of the observed data given the model. The deviance for model 1 is 24185, which is lower than model 2's deviance of 24210. A likelihood ratio test comparing the two models yields a Chi-square value of 25.094 with 1 degree of freedom, which is highly significant (p < 0.001), indicating that the additional quadratic term in model 1 significantly improves the model fit. Thus, model 1 is statistically superior to model 2 in explaining the variation in the importance of veiling.

Models:

model1: veiling ~ importanceOfReligion + incomeLevel + Sex + SizeOfTown + I(SizeOfTown^2) + (1 | country)

model2: veiling ~ importanceOfReligion + incomeLevel + Sex + SizeOfTown + (1 | country)

| **Table S2**. The Fixed and Random Effects for the Model Predicting Importance of Veiling for both Men and Women (*N* = 9,069) Including Sex by Size of Town Interaction | | | | |
| --- | --- | --- | --- | --- |
|  | Estimate | Std. Error | *t* value | *p* value |
| Intercept | 2.67 | 0.19 | 14.05 | < .001*** |
| Importance of Religion | 0.52 | 0.03 | 15.99 | < .001*** |
| Sex (Male) | 0.08 | 0.02 | 2.27 | 0.072 |
| Income Level | 0.00 | 0.00 | 0.36 | 0.732 |
| Size of Town | -0.17 | 0.03 | -6.06 | < .001*** |
| (Size of Town)2 | 0.01 | 0.01 | 4.94 | < .001*** |
| Sex (Male) x Size of Town | 0.00 | 0.00 | -0.94 | 0.344 |
| Random Effects | Variance | Std. Dev. |  |  |
| Intercept (Country) | 0.1 | 0.33 |  |  |
| Residual | 0.84 | 0.91 |  |  |
| Note: * *p* < .05, ** *p* < .01, *** *p* < .001. | | | |  |

| **Table S3.** The Fixed and Random Effects for the Model Predicting Importance of Veiling for both Men and Women (*N* = 7,883) Including Education | | | | |
| --- | --- | --- | --- | --- |
|  | Estimate | Std. Error | *t* value | *p* value |
| Intercept | 2.76 | 0.20 | 13.69 | < .001*** |
| Importance of Religion | 0.53 | 0.03 | 15.40 | < .001*** |
| Income Level | 0.01 | 0.00 | 2.68 | .007 ** |
| Sex (Male) | 0.07 | 0.02 | 3.22 | .001 ** |
| Education | -0.03 | 0.00 | -6.50 | < .001*** |
| Size of Town | -0.19 | 0.03 | -6.36 | < .001*** |
| (Size of Town)2 | 0.02 | 0.00 | 5.18 | < .001*** |
| Random Effects | Variance | Std. Dev. |  |  |
| Intercept (Country) | 0.11 | 0.34 |  |  |
| Residual | 0.86 | 0.93 |  |  |
| Note: * *p* < .05, ** *p* < .01, *** *p* < .001. | | | |  |

| **Table S4.** The Fixed and Random Effects for the Model Predicting Importance of Veiling for Men (*N* = 4,228) Including Education | | | | |
| --- | --- | --- | --- | --- |
|  | Estimate | Std. Error | *t* value | *p* value |
| Intercept | 2.75 | 0.23 | 11.72 | < .001*** |
| Importance of Religion | 0.56 | 0.04 | 12.87 | < .001*** |
| Income Level | 0.02 | 0.01 | 3.13 | .001 ** |
| Education | -0.03 | 0.01 | -4.85 | < .001*** |
| Size of Town | -0.23 | 0.04 | -5.75 | < .001*** |
| (Size of Town)2 | 0.02 | 0.00 | 4.83 | < .001*** |
| Random Effects | Variance | Std. Dev. |  |  |
| Intercept (Country) | 0.11 | 0.33 |  |  |
| Residual | 0.84 | 0.92 |  |  |
| Note: * *p* < .05, ** *p* < .01, *** *p* < .001. | | | |  |

| **Table S5.** The Fixed and Random Effects for the Model Predicting Importance of Veiling for Men (*N* = 3,655) Including Education | | | | |
| --- | --- | --- | --- | --- |
|  | Estimate | Std. Error | *t* value | *p* value |
| Intercept | 2.89 | 0.28 | 10.23 | < .001*** |
| Importance of Religion | 0.48 | 0.06 | 8.61 | < .001*** |
| Income Level | 0.00 | 0.01 | 0.42 | 0.671 |
| Education | -0.03 | 0.01 | -4.42 | < .001*** |
| Size of Town | -0.14 | 0.05 | -3.03 | .002 ** |
| (Size of Town)2 | 0.01 | 0.00 | 2.33 | .019 * |
| Random Effects | Variance | Std. Dev. |  |  |
| Intercept (Country) | 0.12 | 0.35 |  |  |
| Residual | 0.87 | 0.93 |  |  |
| Note: * *p* < .05, ** *p* < .01, *** *p* < .001. | | | |  |
